# Facilitating engagement with PrEP and other HIV prevention technologies through practice‐based combination prevention

**DOI:** 10.1002/jia2.25294

**Published:** 2019-07-22

**Authors:** Morten Skovdal

**Affiliations:** ^1^ Department of Public Health University of Copenhagen Copenhagen Denmark

**Keywords:** HIV prevention, combination prevention, innovations, HIV prevention technologies, PrEP, social practice theory

## Abstract

**Introduction:**

Recent years have witnessed a rapid expansion of efficacious biomedical HIV prevention technologies. Promising as they may be, they are largely delivered through standard, clinic‐based models, often in isolation from structural and behavioural interventions. This contributes to varied, and often poor, uptake and adherence. There is a critical need to develop analytical tools that can advance our understandings and responses to the combination of interventions that affect engagement with HIV prevention technologies. This commentary makes a call for practice‐based combination HIV prevention analysis and action, and presents a tool to facilitate this challenging but crucial endeavour.

**Discussion:**

Models and frameworks for combination HIV prevention already exist, but the process of identifying precisely what multi‐level factors that need to be considered as part of a combination of HIV interventions for particular populations and settings is unclear. Drawing on contemporary social practice theory, this paper develops a “table of questioning” to help interrogate the chain and combination of multi‐level factors that shape engagement with HIV prevention technologies. The tool also supports an examination of other shared social practices, which at different levels, and in different ways, affect engagement with HIV prevention technologies. It facilitates an analysis of the range of factors and social practices that need to be synchronized in order to establish engagement with HIV prevention technologies as a possible and desirable thing to do. Such analysis can help uncover local hitherto un‐identified issues and provide a platform for novel synergistic approaches for action that are not otherwise obvious. The tool is discussed in relation to PrEP among adolescent girls and young women in sub‐Saharan Africa.

**Conclusions:**

By treating engagement with HIV prevention technologies as a social practice and site of analysis and public health action, HIV prevention service planners and evaluators can identify and respond to the combination of factors and social practices that interact to form the context that supports or prohibits engagement with HIV prevention technologies for particular populations.

## 1 Introduction

Despite some successes in HIV prevention, 1.8 million people were infected with HIV in 2017, and rates of infection grew in more than 50 countries 1. In sub‐Saharan Africa, the region worst affected by the HIV epidemic, more than a third of new infections in 2017 occurred among young people (15 to 24 years) 1. Although adolescent girls and young women (15 to 24) only make up 10% of the population in sub‐Saharan Africa, they account for a quarter of all new HIV infections 2. This, coupled with a so‐called “youth bulge” in sub‐Saharan Africa 3, has contributed to a sense of urgency to harness recent biomedical and health service successes in HIV treatment and rapidly expand the availability of biomedical HIV prevention technologies.

Promising as these innovations may be, biomedical HIV prevention technologies are largely implemented in isolation from structural and behavioural interventions, with little recognition of their synergies 4. The difficulty of identifying the combination of biomedical, structural and behavioural interventions required for strategic advantage and synergy is widely recognized 4, 5, 6. This challenge is compounded by the absence of an analytical framework to help HIV prevention service planners and evaluators identify the combination of interventions that work, under what circumstances, for whom, and with which HIV prevention practices in focus. This commentary has two aims. One, to argue that the biomedical turn in HIV prevention presents both a need and an opportunity to conceptualize practice‐based combination prevention as an approach for disentangling and responding to the range of behavioural, biomedical and structural elements that interact with non‐linear and multiplying effects to shape HIV prevention practices, including engagement with HIV prevention technologies. Two, to develop and demonstrate a tool for interrogating and responding to the chain, sequence or combination of factors that affect engagement (or otherwise) with HIV prevention technologies, as well as the role of other shared social practices.

## 2 Discussion

### 2.1 The biomedical turn in HIV prevention

Remarkable progress has been made in expanding the portfolio of biomedical HIV prevention technologies available to young people. We now know that people living with HIV (PLHIV) and on antiretroviral therapy can reach undetectable levels of viral load, which prevents them from transmitting HIV to their sexual partners 7. This is referred to as treatment as prevention (TasP). Antiretroviral drugs can also be taken orally by HIV negative people as a pre‐exposure prophylaxis (PrEP) or post‐exposure prophylaxis (PEP), significantly reducing the risk of becoming infected 8, 9. Alternative ways of delivering antiretroviral drugs, such as through vaginal rings 10, microbicide gels 11 or films 12, are being tested in demonstration projects. Voluntary medical male circumcision (VMMC) has proved efficacious, lowering men's risk of HIV infection by up to 60% 13. These technologies, with the exception of VMMC, are considered “highly user‐dependent” and adherence is repeatedly stated as the strongest determinant of their effectiveness 14, 15.

Unfortunately, examples from across the globe highlight varied uptake and adherence to these HIV prevention technologies, particularly among young people 2, 16. While uptake and adherence to PrEP is generally high among certain groups of men who have sex with men in high‐income settings 17, disappointing levels of uptake and adherence to PrEP among adolescent girls and young women (AGYW) is widespread in sub‐Saharan Africa 18, 19. Systematic reviews have found PEP adherence to be generally poor, but particularly so among adolescents 20. Uptake of VMMC continues to be slow in a number of sub‐Saharan African countries 21, although some countries, like South Africa 22, have witnessed rapid increases of uptake in recent years. Successes in VMMC scale‐up, however, are often attributed to school‐based programmes targeting males 10 to 14, with young men falling behind 23. While treatment as prevention has demonstrated its effectiveness, emerging evidence from South Africa suggest that poor levels of antiretroviral drug adherence among sexually active adolescents living with HIV may undermine treatment as a form of secondary prevention 24. These varied outcomes suggest that populations and settings appropriate and respond to HIV prevention technologies in different ways. To optimize engagement with HIV prevention technologies, we need to meet young people where they are 25, and uncover local hitherto un‐identified issues that obstruct their uptake and engagement with these technologies, and identify novel approaches for action that are not otherwise obvious.

Current emphasis on biomedical HIV prevention technologies confronts HIV prevention service planners and evaluators with two challenges. One, to refrain from falling into the trap of assuming that individuals are capable of making informed, rational and unfettered choices for themselves, renewing emphasis on the behaviour of individuals to make use of, and consistently adhere to, HIV prevention technologies. Two, to consider how a broader set of political, social, cultural and ethical issues interact to shape the ability and decision of young people to engage or disengage with HIV prevention technologies. Herein lies the opportunity for a more focused and practice‐oriented approach to combination HIV prevention. Rather than utilizing standard, clinic‐based models to promote uptake and adherence – assuming young people to be observers of specific behaviours following recommendations from a healthcare provider – there is a need to recognize the broader set of factors and other shared social practices (structural elements) that need to be synchronized to shape engagement (behavioural elements) with HIV prevention technologies (biomedical elements). Practice‐based combination prevention, in an era of biomedical HIV prevention, is therefore about identifying and responding to the combination of multi‐level factors and other shared social practices whose synergies create the context for particular populations that supports or prohibits the practice of engaging with HIV prevention technologies.

### 2.2 Existing combination HIV prevention models and frameworks

A few existing frameworks and models for combination HIV prevention do exist, and include among others the Multiple Domain Model 26, the Dynamic Social Systems Model 27, the Network‐Individual‐Resource Model 28, the HIV prevention cascade 29, 30, 31 and Complex Systems theory 4, 32. Each of these models and frameworks usefully highlight how a range of factors influence each other in complex ways, with implications for how individuals effectively deploy HIV prevention technologies or behaviours. Useful as they are, the process of identifying precisely what multi‐level factors that need to be considered as part of a combination HIV prevention intervention for particular HIV prevention practices, populations, settings and stages of the epidemic is unclear. Interventions are often selected based on available evidence, but key factors may be missed if HIV prevention service planners and evaluators primarily draw on published evidence from other contexts. Furthermore, pinpointing exactly how structure and the social intersect with individual behaviour, affecting HIV prevention, is difficult and complex, hampering both empirical research and combination prevention interventions. This challenge has been noted by Susan Kippax 33 who warns against HIV research and public health models that identify structural factors without interrogating the mediating links between individual, community and societal phenomena. Kippax 34 posits that it is through social practices we can understand and shape the relationship between multiple levels of influence. For these reasons, I look to contemporary theories of practice and draw on the vocabulary they have developed to propose a tool for analysing and understanding how structure, individual behaviour and biomedical technologies interact and come together to affect engagement with HIV prevention technologies. The tool supplements existing models and frameworks for combination HIV prevention by sparking conversations and local research to uncover new issues and connections between defining factors, which may lead to specific actions.

### 2.3 A “table of questioning” tool for practice‐based combination HIV prevention

Reckwitz [35: p. 249] defines a practice as “[a] routinized type of behaviour which consists of several elements, interconnected to one other: forms of bodily activities, forms of mental activities, ‘things’ and their use, a background knowledge in the form of understanding, know‐how, states of emotion and motivational knowledge.” According to this definition, agency and the routine practice of engaging with HIV prevention technologies is enabled by numerous overlapping factors of influence coming together. These different factors form part of the fabric of our everyday lives, across scales from the individual to the macro as well as space and time. The factors consist of, or give rise to, a broad domain of human activities, which both reproduce or transform the factors themselves and social practices that overlap to coordinate and synchronize the practice of engaging (or otherwise) with HIV prevention technologies. According to Blue et al. 36, looking at public health practices is critical if health service planners and evaluators are to disentangle the configuration of factors, or hybrid of social practices, which establish healthy practices as (im)possible or (un)desirable.

Drawing on the work of contemporary social practice theorists, including Kemmis et al. 37 and Shove et al. 38, the proposed “table of questioning” offers a strategy for facilitating analysis and action for practice‐based combination HIV prevention. The tool presents 10 questions, which in a two‐step process seek to facilitate reflection and analysis of the range of factors that shape (dis)engagement with a particular HIV prevention technology. The tool is by no means all encompassing. It merely provides a flavour of how placing emphasis on the practice of (dis)engaging with HIV prevention technologies can offer new insight and direction for practice‐based combination HIV prevention. Practices vary in scope and size, and the boundaries (type of technology, setting, population group, timing), while permeable, should be established by the objectives of the analysts 36.

**Figure 1:**
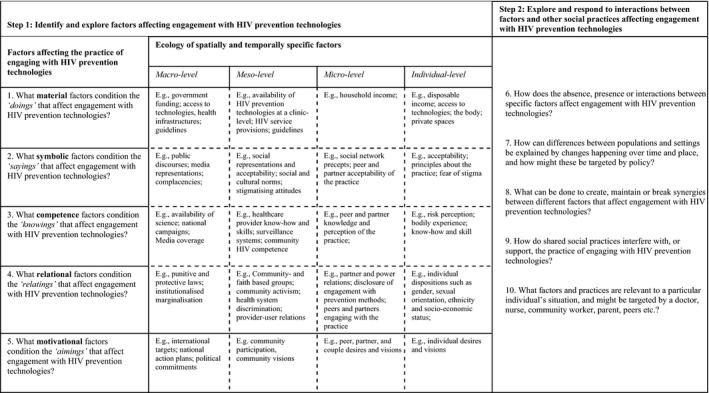
A “table of questioning” for practice‐based combination HIV prevention.

The tool presents a matrix with five different types of factors across four socio‐ecological levels (see Figure 1). The four ecological levels, namely “macro,” “meso,” “micro” and individual levels, akin to Bronfenbrenner's socio‐ecological framework 39, have been plotted into the tool in response to a call for greater clarity of how practices, enacted by people at a micro‐level, are positioned in macro structures 40. The tool encourages HIV prevention service planners and evaluators to first explore the constellation of factors that affect engagement with HIV prevention technologies for a particular population in a particular setting (step 1). Insights from this step can then be used to interrogate links and connections between the factors, and examine how these synergies, often in interaction with other shared social practices, shape the ability or desire for specific population groups to engage, or disengage, with HIV prevention technologies (step 2). To facilitate the exploration, both steps lists a series of questions. The questions have been formulated to spark conversation about how a practice, such as engagement with HIV prevention technologies, either emerges, persists or disappears. Reflecting the work of Blue et al. 36 and Shove et al. 38, the questions allow analysts to explore how best to make or break links between defining factors; understand the competition and collaboration that exists between the factors and associated practices; and develop insight into how practitioners get recruited, maintained or defected from the social practice under scrutiny. By asking these questions, HIV prevention service planners and evaluators will be able to explore, explain and respond to differences in practice between people and settings. Practically, the tool can be used figuratively to tabulate the range of factors and social practices associated with engagement with a particular HIV prevention technology, or one can draw on the questions in a variety of formats and fora to instigate reflection and analysis, with the matrix visually reminding the analysts to consider different dimensions and levels of analysis.

### 2.4 Practice‐based combination HIV prevention for PrEP among AGYW in sub‐Saharan Africa

As exemplified by the DREAMS programme 43, PrEP increasingly forms part of the expanding portfolio of interventions being made available to AGYW in sub‐Saharan Africa to prevent HIV acquisition 41, 42. The DREAMS programme represents a breakthrough in HIV prevention in sub‐Saharan Africa, by laudably availing layers of quality and evidence‐informed interventions, covering biomedical, structural and behavioural initiatives. Saul et al. [43: p. 12], in describing the potential of DREAMS, argue that “meeting the needs and demands of AGYW requires unpacking the data to identify challenges and risks for an individual girl or young woman. Once identified, then and only then, can a response be tailored to mitigate risks in a holistic way.” Rather than implementing structural, behavioural and biomedical interventions in isolation, the DREAMS programme allow service providers to target AGYW with a number of interventions. Promising as this may be, it is unclear what constitutes “data,” and what frameworks are used to ascertain which interventions to offer particular AGYW, and with what combination‐synergies. Furthermore, the DREAMS programme focuses on creating an enabling environment for HIV prevention, but pays less attention to the motivation of AGYW to engage with HIV prevention practices routinely. While comprehensive, the DREAMS programme is not exhaustive, and it is likely that local material, symbolic, competence, relational and motivational factors, and associated social practices that affect AGYW motivation and capacity to engage with PrEP have not been considered. This may either be because “data” are not available, or because the core package interventions focus on “what works” (evidence‐informed programming), as opposed to how the interventions work in a given context, for whom, and with what interactions to achieve strategic advantage and synergy 4.

The proposed “table of questioning” can help HIV prevention programme planners and evaluators hone in on local determining factors and social practices. This can help them uncover, monitor and respond to the constellation of factors, and related social practices that affect engagement with PrEP. In the case of PrEP, this is important for a number of reasons. While PrEP can reduce risk of HIV by over 90% when taken consistently 8, PrEP trials with African women have found adherence levels so low, particularly among young women, that efficacy could not be ascertained 18, 19. Commentators highlight numerous demand‐side, supply‐side and adherence barriers 25, 41, 44, warranting urgent attention to constellations of factors and social practices that recruit and maintain AGYW as “engagers” with PrEP. In other words, PrEP should not merely be seen as a biomedical intervention to be included in a combination of HIV preventions, but recognized as a practice that is contingent on the configuration of multi‐level factors that establish engagement with PrEP as (im)possible and (un)desirable. Haberer et al. 25 argue that we must consider the broad range of local factors that make PrEP both relevant, appealing and available to AGYW. The proposed “table of questioning” can help HIV prevention service planners and evaluators identify what those factors and social practices may be. Specifically, the “table of questioning” can help disentangle how the presence or absence of material, symbolic, competence, relational, and motivational factors, either enable or constrain the array of activities, or “doings,” “sayings,” “knowings,” “relatings” and “aimings” that affect AGYWs engagement with PrEP. What may some of these factors be?

If the public or AGYW brand PrEP as a “promiscuity pill” 45, or consider PrEP ineffective and inappropriate for dissemination 46, this may limit demand for PrEP. Knowledge and awareness of HIV risk is a defining element of willingness to engage with PrEP, yet, a Zimbabwean study has found that many young people at increased infection risk did not perceive to be at high risk 47. Relatedly, the FEM‐PrEP study found women to underestimate their risk of infection and that perceived risk was associated with greater engagement with PrEP 48. Social relations and partner relations also matter. If male partners, in contexts of male dominance, are not supportive of their partners using PrEP, this may prevent some AGYW from engaging with PrEP 49. Similarly, HIV prevention service providers may not be supportive of AGYW seeking PrEP because of attitudes towards adolescent sexuality, and concerns about behavioural disinhibition due to PrEP 50. In terms of motivational factors, a study in South Africa found that a personal desire for HIV protection, and a wish to keep engagement with a HIV prevention technology a secret, positively affected demand for PrEP 51. Among HIV‐uninfected Kenyan women in serodiscordant relationships, the desire to remain HIV uninfected and have a HIV‐free infant have also been found to motivate uptake and continued use of PrEP during pregnancy 52.

Haberer et al. 25 note that adolescents and young people live social and connected lives, which are characterized by their quest for novelty and sensation. The everyday practices of young people inevitably intersect with engagement with PrEP. Scorgie et al. 53, for instance, have found both the timing and location of young South African women's sexual intimacy to be unpredictable, making engagement with on‐demand oral PrEP a challenge. These factors not only differ significantly from setting to setting, explaining varied engagement with PrEP, but also interact in complex ways. If a setting experiences drug stock‐outs (perhaps due to cuts in funding), this may not only remove a defining material factor, but also negatively impact PrEP users trust in health services, and acceptability of the prevention method. Comparing and contrasting scenarios where AGYW or other population groups at risk are either able or unable to engage with PrEP can reveal differences in the composition of factors and social practices that establish engagement with PrEP as (im)possible and (un)desirable. Such analysis can highlight the missing links, and the actions required to avail the factors and practices that support engagement with PrEP. For instance, if differences in the uptake of PrEP within a country can partly be explained by differences in healthcare provider PrEP awareness, familiarity, comfort and prescribing experiences 54, this could constitute a missing link and avenue for action.

## 3 Conclusions

Practice‐based combination prevention treats HIV prevention practices as sites of analysis and public health action. Taking a practice‐oriented approach to combination prevention enables HIV prevention service planners and evaluators to recognize and consider the range of factors, social practices and interventions that need to be synchronized in order to establish engagement with HIV prevention technologies as a desirable thing to do for particular groups of people, in specific settings. It is a particular pertinent approach in an era of biomedical disease prevention, where the concept of “adherence” has locked us into a narrow understanding of medicine taking and disease self‐management. Rather, practice‐based combination prevention calls for recognition and greater understanding of the range and combination of factors that establish (dis)engagement with HIV prevention technologies as (un)desirable and (im)possible. It also draws attention to the role of other social practices that are associated with engagement with HIV prevention technologies. Given the low rates of PrEP uptake and adherence among AGYW in sub‐Saharan Africa, the proposed “table of questioning” provides a much needed framework and vocabulary to support HIV prevention service planners and evaluators identify system‐synergies 4 for PrEP engagement, which may not otherwise be obvious.

The proposed “table of questioning” cautions against once‐size‐fits‐all responses, recognizing the complex realities of people and differences in the cultural, political and socioeconomic fabric of different settings. However, some factors or interventions may well be applicable and generalizable to different population groups. Differentiating between global (general) and local (specific) factors on the ecology continuum, may reveal macro‐level factors that apply to a large number of population groups, while meso‐, micro‐ and individual‐level factors may require more localized responses. The tool highlights the roles of different stakeholders in making or breaking links to establish engagement with HIV prevention technologies as desirable and possible. Practice‐based combination prevention thus allow us to go beyond the biomedical differentiated care agenda, and work towards differentiated combination preventions. However, first, operational research and evaluations applying and validating this tool to different contexts is urgently needed.

## Competing interest

The author has no conflicts of interest to declare.
